# Impact of haemoglobin variants on the use of haemoglobin A1c for the diagnosis and monitoring of diabetes: a contextualised review

**DOI:** 10.1007/s11845-022-02967-2

**Published:** 2022-04-01

**Authors:** Anne Marie Liddy, Stephan Grundy, Seamus Sreenan, William Tormey

**Affiliations:** 1 Department of Diabetes and Endocrinology, Royal College of Surgeons in Ireland, Dublin, Ireland; 2 Department of Diabetes and Endocrinology, Connolly Hospital, Dublin 15, Blanchardstown, Ireland; 3 Department of Chemical Pathology, Beaumont Hospital, Dublin, Ireland; 4 Biomedical Sciences, University of Ulster, Coleraine, Northern Ireland

**Keywords:** Diabetes, Diagnosis, HbA1c, Haemoglobinopathy

## Abstract

HbA1c is the established test for monitoring glycaemic control in diabetes, and intervention trials studying the impact of treatment on glycaemic control and risk of complications focus predominantly on this parameter in terms of evaluating the glycaemic outcomes. It is also the main parameter used when targets for control are being individualised, and more recently, it has been used for the diagnosis of type 2 diabetes. For laboratories performing this test and clinicians utilising it in their decision-making process, a thorough understanding of factors that can impact on the accuracy, and appropriate interpretation of the test is essential. The changing demographic in the Irish population over the last two decades has brought this issue sharply into focus. It is therefore timely to review the utility, performance and interpretation of the HbA1c test to highlight factors impacting on the results, specifically the impact of haemoglobin variants, and the impact of these factors on its utilisation in clinical practice.

## Introduction

Over the last couple of decades, the haemoglobin A1c (HbA1c) has become firmly established as the primary indicator of glycaemic control in diabetes thanks to the evidence base from trials such as the Diabetes Control and Complications Trial and the UK Prospective Diabetes Study [1, 2]. More recently, HbA1c has become accepted as a suitable test for the diagnosis of type 2 diabetes [3]. The broadening of the indication for its use and the efforts to standardise testing internationally have also re-focused attention on the limitations of the HbA1c in clinical practice. The implications of a diagnosis of diabetes—not least the fact that in Ireland, it means that the cost of all diabetes-related treatment is covered by the public purse—mean that an understanding of the limitations of the test, for example, the impact of haemoglobin variants on the level, is important for those ordering and interpreting it.

The changing demographic of the Irish population over the last 15 years brings this point into sharp focus. Up to the 1990s, the Republic of Ireland was a very homogenous population. However in the 2016 census, of a total population of 4,761,865, 11.6% of the population identified as non-nationals [4]. The International Diabetes Federation 2014 prevalence figures for diabetes estimated a prevalence of 8.6% for India, 5.9% for the Philippines, 7.1% in Poland, 4.6% in Nigeria, 8.4% in South Africa and 6.4% in Ireland. For the environment impacts on diabetes prevalence, populations that are at particular risk are likely to retain that risk after migration [5]. In using the HbA1c for diagnosis in Ireland, clearly, it is important to be able to correctly interpret the results of the test.

### HbA1c in the biochemical diagnosis of diabetes

Despite its variability, measurement of glucose has, for decades, been the accepted standard for making a diagnosis of diabetes. The biochemical criteria for the diagnosis of diabetes and dysglycaemia are contained in Table 1. In addition, a random venous plasma glucose ≥ 11.1 mmol/L together with typical symptoms of diabetes can be used as a diagnostic criterion.

**Table 1:** Diagnostic criteria for diabetes [6]

	Fasting glucose (mmol/L)	2-h glucose^a^ (mmol/L)
Normal glucose tolerance	< 5.6	< 7.8
Impaired fasting glucose Impaired glucose tolerance	≥ 5.6, < 7.0	≥ 7.8, < 11.1
Diabetes mellitus	≥ 7.0	≥ 11.1

^a^Two hours after consumption of 75 g anhydrous glucose in an oral glucose tolerance test (OGTT)

In 2011, the World Health Organization (WHO) added HbA1C to these diagnostic criteria. An HbA1c of 6.5% (48 mmol/mol) was defined as the cut-off point for diagnosing diabetes. HbA1c of < 6.5% (48 mmol/mol) does not exclude diabetes defined by glucose testing. The WHO made no recommendation on HbA1c below 6.5% (48 mmol/mol) because of the lack of evidence [7].

HbA1c between 6.0% (42 mmol/mol) and 6.5% (48 mmol/mol) should be considered for diabetes prevention [7]. The American Diabetes Association suggests HbA1c 5.7% (39 mmol/mol) to 6.4% (46 mmol/mol) as the high risk range [3]. In an asymptomatic person, the diagnosis of diabetes should not be made on the basis of a single abnormal plasma glucose or HbA1c value. At least one other abnormal HbA1c or plasma glucose value is needed from any one of a fasting, random or OGTT sample to make the diagnosis.

Our previous work has shown discordance in the diagnosis of diabetes when the glucose and HbA1c criteria are used to make the diagnosis. We reviewed the results of our experience with OGTT over a 4 and a half year period between 2002 and 2006. Of the total of 312 OGTTs performed in that time, 118 (37.8%) were normal, 94 (30.1%) had diabetes, and the remainder had impaired fasting glucose and/or impaired glucose tolerance (Table 2) [8].

**Table 2:** Fasting plasma glucose (FPG) with corresponding 2-h glucose levels from 312 OGTTs

FPG (mmol/l)	Normal 2-h glucose (*n* = 172)	2-h glucose: IGT (*n* = 68)	2-h glucose: diabetes (*n* = 72)
< 5.6 (*n* = 137)	118 (86.1%)	12 (8.8%)	7 (5.1%)
≥ 5.6, < 6.1 (*n* = 45)	22 (48.9%)	18 (40%)	5 (11.1%)
≥ 6.1, < 7.0 (*n* = 65)	26 (40%)	22 (33.8%)	17 (26.2%)
≥ 7.0 (*n* = 65)	6 (9.2%)	16 (24.6%)	43 (66.2%)

*OGTT* oral glucose tolerance test, *IGT* impaired glucose tolerance

HbA1c data were also available on 185 of 312 (59%) subjects including 64 patients with diabetes defined by traditional glucose criteria (mean HbA1c 6.6 ± 0.7% [49 ± 8 mmol/mol] compared to 5.4 ± 0.5 [36 ± 6 mmol/mol] in subjects with normal glucose tolerance, *p* < 0.001). Among subjects with diabetes by traditional criteria, HbA1c was < 6.5% (48 mmol/mol) in 29 (31%) and < 6% (42 mmol/mol) in 10 (11%). There were 12 subjects who had an HbA1c > 6.5% (48 mmol/mol) who did not meet the traditional diagnostic criteria for diabetes. Of these, 6 had IFG, 4 had IFG/IGT, and 2 had normal glucose tolerance [8]. These data, highlighting the discordance between glucose and A1c when diagnosing diabetes, serve to remind us of the importance of understanding factors that can influence the HbA1c.

The use of HbA1c requires stringent quality assurance and assays standardised to the International Federation for Clinical Chemistry (IFCC) reference values. While the test can be utilised for monitoring diabetes control even in the setting of factors that might interfere with the results as long as those factors are taken into account, we feel that the use of HbA1c for the diagnosis of diabetes requires greater stringency, given the implications of the diagnosis. Ideally there should be no conditions present which preclude accurate measurement.

Annex 1 of the WHO 2011 report lists some of the factors that influence HbA1c levels and measurement [7, 9]. Factors include those that can impact on red cell lifespan (such as haemolysis which can shorten survival and lower the HbA1c or iron deficiency which can lengthen survival and increase the HbA1c) and carbamylation of haemoglobin in patients with renal failure. The *Canadian Journal of Diabetes* offers a succinct summary of current practice [10]. An important factor to bear in mind in the interpretation of an HbA1c result is the possible presence of an abnormal haemoglobin or “haemoglobinopathy”.

### Haemoglobin physiology

Haemoglobin is a globular molecule containing four subunits. Each of the four subunits contains a heme porphyrin derivative. There are two pairs of polypeptides in each haemoglobin molecule which constitute the globin portion of haemoglobin. In normal adult haemoglobin HbA (or HbA1), there are two pairs of polypeptides called the α chain with 141 amino acids and the β chain with 146 amino acids (HbA has α2 β2 chains). About 2.5% of haemoglobin in normal adults is HbA2 in which β chains are replaced by δ chains. The δ chain also contains 146 amino acids but differs by 10 residues from the β chain.

Foetal haemoglobin (HbF) is similar to HbA, but the β chains are replaced by ϒ chains (HbF is α2ϒ2). The two α subunits and the two β subunits are identical to each other, but when the Hb tetramer is formed, the two α subunits differ slightly in their contacts with the β subunits. The α1 and β1 associate as a dimer as do the α2 and β2 chains. These are relevant to oxygen carriage [11].

The formation of dimers between α and β chains is driven by electrostatic forces with positively charged α chains and negatively charged β chains. β-globin variants usually acquire a positive charge which slows the assembly of haemoglobin. δ-chains are also positively charged with little affinity for α chains. Some of the functional clinical effects are listed in Henry’s textbook [12]. When the protein component of the haemoglobin molecule is defective or deficient, the resulting abnormal haemoglobins, or “haemoglobinopathies”, can have clinical effects and impact on interpretation of HbA1c.

### Haemoglobinopathies

The major types of inherited disorders affecting haemoglobin are the haemoglobin variants in which abnormal polypeptide chains are produced and thalassaemias in which the chains are normal in structure but insufficiently produced because of globin gene deletions or a mutation rendering the globin genes non-functional.

Haemoglobin variants may have variable effects on the measurement of HbA1c. There are 1,198 haemoglobin variants described to date [13], and many are clinically silent. The haemoglobin variants that are most clinically relevant include haemoglobins S (sickle cell), C and E which are due to point mutations leading to amino acid substitutions in the globin portion of the molecule. Depending on the number of alleles of the gene that are abnormal, combinations of different haemoglobins may be found, and this can colour the clinical picture. For example, HbS has normal α chains but abnormal β chains. There is a valine for glutamic acid substitution in position 6 on the β polypeptide chain. At least 5.2% of the human population carries a clinically significant haemoglobin variant [14]. This is an important issue in the West Dublin catchment area due to the high numbers of individuals from malaria areas which have a high prevalence of the sickle cell trait. We recently reviewed all of the HbA1cs measured in our laboratory over a 6-month period (*n* = 3920). Of these, 28 patients (0.71%) had an abnormality detected suggesting the presence of an abnormal haemoglobin. Nine patients had haemoglobin electrophoresis performed demonstrating the presence in each case of a pattern consistent with sickle cell trait. General practitioners, diabetologists, their teams and hospital laboratories should be cautious in using HbA1c alone for the diagnosis of diabetes in this context and should take appropriate steps to limit the potential misuse of HbA1c in this setting and offer an alternative if an abnormal haemoglobin band is demonstrated when measuring HbA1c. If discordance is seen with OGTT’s with HBA1c, consideration of the presence of a haemoglobin variant should be considered.

### HbA1c

Total glycated haemoglobin comprises all of the haemoglobin that has been glycosylated at one or more amino acids in either the α or β chain in the globin portion. HbA1c is defined by the IFCC as haemoglobin that is irreversibly glycated at one or both N-terminal valines of the β chains of haemoglobin and accounts for 80% of glycated haemoglobin. Glycation occurs in two steps. Firstly, a labile Schiff base is formed followed by an irreversible non-enzymatic Amadori rearrangement. The HbA1c with HbA1b and HbA1a are the fast migrating components of HbA on gel electrophoresis. The remaining glycated haemoglobins have glucose, glucose-6-phosphate, fructose-1–6-diphosphate or pyruvic acid bound to 1 of 44 additional sites at ε-amino groups of lysine residues or at the NH_2_ terminus of the α chain. Detection of non-A1c glycated haemoglobin varies across available methods [15].

## Laboratory measurement of HbA1c and technical factors impacting on interpretation

### HbA1c and IFCC

In 1994, the IFCC defined HbA1c as “the HbA1 glycated at the N-terminal amino acid group of the β chain”. A reference method was developed, and a network of reference laboratories was set up. The reference method is a specific enzymatic cleavage of the glycated and non-glycated Hb resulting in a glycated and non-glycated β-N-terminal hexapeptide. The hexapeptides are first separated from the remaining peptides by reverse-phase HPLC and then quantified by either capillary electrophoresis or electron spray ionisation mass spectrometry [16]. Because the reference procedure provided a methodological anchor for HbA1c analysis, a calculation was agreed to align the new values with Diabetes Control and Complications Trial (DCCT) values [17]. IFCC values are used in the European Union national HbA1c standardisation schemes.

### Conventional current methods

One of four methods is usually employed in HbA1c estimations: (a) HbA1c specific immunoassays; (b) ion-exchange HPLC; (c) boronate affinity HPLC methods; and (d) a direct enzymatic method. Furthermore, capillary electrophoresis systems such as the Sebia Capillarys 2 Flex Piercing system claim to have no interferences from carbamylation, bilirubin, triglycerides and haemoglobin variants such as S, D, C and E or beta-thalassemia samples. It satisfies the IFCC and National Glycohaemoglobin Standardisation Program (NGSP) guidelines [18]. A multicentre evaluation proved its suitability for routine clinical use [19]. Currently, ion-exchange HPLC, boronate affinity HPLC methods and immunoassays are the commonest methods used to estimate HbA1c. One of the factors that can interfere with the interpretation of these results of HbA1c is a haemoglobin variant.

Haemoglobin variants have diverse effects on the measurement of HbA1c. The complexity of the identification of these haemaglobin variants was critically reviewed [20]. Methods for high-throughput screening include HPLC and capillary zone electrophoresis. There are practical problems in rare cases because migration patterns using these two methods are not completely specific. The American College of Pathology requires all laboratories providing haemoglobin analysis to provide a secondary method to confirm variants found by primary screening methods. Isoelectric focusing with gel electrophoresis, mass spectrometry and molecular diagnostics may form parts of the Hb variant identification process [20] In reference laboratories, electrospray mass spectrometry can be used to identify more than 700 haemoglobin variants but is outside the menu of routine service laboratories [21].

### What is the “best” method

The boronate affinity HPLC methods appear to suffer least from haemoglobin variant interference. The report on the ion-exchange GX analyser for HbA1c in a Japanese population demonstrates the superiority of boronate methods. HbD, HbS and HbC had HbA1c values close to that of boronate affinity and immunoassay methods, but HbA1c values with HbE or other variants that elute before or nearly at the same time as HbA0 were lower [22].

Recent data show that commercial analyser performances are fit for stringent clinical purposes [23]. In boronate affinity methods, m-aminophenylboronic acid reacts specifically with cis-diol groups on glucose which is bound to haemoglobin. Boronate affinity measures the ratio of total glycated to non-glycated haemoglobin irrespective of the haemoglobin variant. There is interference with high levels of HbF due to the lower glycation rate of HbF compared to HbA. In that circumstance, there will be a false low HbA1c value [24]. Hydroxyurea is used in the treatment of cancer as well as sickle cell disease, as it has been shown to raise HbF levels. Therefore, patients who are being treated with hydroxyurea may have an artificially low HbA1c level [25].

A contrary view is that in areas of high prevalence of Hb variants, that methods such as capillary electrophoresis that identify presumptive Hb variants should be preferred over immunoassay or boronate affinity chromatography [26].

Market research forecasts to 2019 predict that boronate affinity chromatography devices that offer the least interference from Hb variants in HbA1c testing will likely dominate the testing devices market [27].

### HA-8160 as a tool to analyse HbA1c

The HA-8160 is our routine analyser to measure HbA1c. It is a reverse-phase cation-exchange HPLC from Akray-Adams (Menarini) which is fully automated. Cation-exchange HPLC separates haemoglobin fractions based on charge differences. It uses colorimetric detection at 415 nm and a blanking wavelength of 500 nm. The results are presented as %HbA1c, %HbA and %HbF. These are calculated from the peak areas of different haemoglobin fractions as a % of total haemoglobin according to the equation [28]:

$${\%}{HbA1c} = {100} \times {HbA1c/HbA + HbA1c}$$

Thus, variant Hbs or Hb adducts or derivatives that elute separately from HbA and HbA1c have little effect on the calculation above [28].

Haemoglobin variants, however, can have variable effects on HbA1c using this method. Variant haemoglobin (X) forms may affect the %HbA1c by (a) HbX co-eluting with the HbA1c moiety to massively falsely increase the proportion value; (b) HbX1c co-eluting with HbA1c but the HbX separates from the HbA causing an apparent but false increase in HbA1c; and (c) HbX co-eluting with HbA but HbX1c separates from the elution of HbA1c causing a false reduction in HbA1c. Examples of each variation are listed [29].

Published figures for HA-8160 showed that between batch imprecisions were 1.58% and 0.88%, respectively, at a mean level of HbA1c of 5.9% (40 mmol/mol) and 11% (97 mmol/mol). The study showed a negative interference of − 0.1% for HbAC and − 0.4% from interference by HbS [14]. The reported decrement with 3% carbamylated Hb was − 0.2% [30]. Listings of method interferences by the NGSP to September 2014 list no interference with HA 1980 from HbC, HbS or from an HbF < 30% [31]. In a comparison with the “Primus” boronate affinity HPLC method (ultra), the HA-8160 in diabetes mode showed a reduction of − 0.82% at HbA1c of 6% (42 mmol/mol) and a reduction of − 1.14% at a HbA1c of 9% (75 mmol/mol) with HbE trait and a reduction due to HbD trait of − 2.17% at HbA1c of 6% (42 mmol/mol) and of − 1.58% at HbA1c of 9% (75 mmol/mol). Using the HA-8160 TP mode, the effects of HbE trait were a reduction of − 0.14% at HbA1c of 6% (42 mmol/mol) and − 0.28% at HbA1c of 9% (75 mmol/mol) [32]. Roberts found little effect of HbS (HbAS) or HbC trait using HA-8160. For HbS trait, the effects were − 0.24 and − 0.83 at 6% (42 mmol/mol) and 9% (75 mmol/mol) HbA1c, respectively, while for HbC, the effects were + 0.02% and 0.22% at the same HbA1c levels [33]. A patient with homozygous HbSS had no detectable HbA1c by HPLC [34]. Another case reported an undetectable HbA1c by HA-8160 but a value of 6.1 (43 mmol/mol) to 7.3% (56 mmol/mol) in the same 65-year-old female type 2 patient with diabetes with HbD Punjab when measured immunoturbidimetrically by Cobas Integra (Roche Diagnostics) [35]. In a comparison study of 27 patients with HbD Punjab (also known as HbD Los Angeles) heterozygotes, using HA-8160, there was no result in 16 cases and false low in the 11 others when compared with the Tina-quant HbA1c Gen 3 turbidimetric immunoassay with a Cobas 6000 (Roche Diagnostics) [36]. The use of the high-resolution program on the HA-8160 also identified interference in HbA1c determination by clinically silent variants Hb Graz, Hb Sherwood Forest, Hb O Padova and HbD [31]. Where the haemoglobin variant carrier has an acetylated NH_2_ terminus with Hb Raleigh, Hb Long Island and HbA2 Niigata, Hb glycation cannot be used for glycaemic monitoring [37]. Therefore, awareness of abnormal haemoglobins when testing for HbA1c is of major importance.

## HbA1c in a more global context

Factors which may play a role in variation of HbA1c values may include different red cell survival as suggested based on data from the Diabetes Prevention Program [38]; female sex [39]; sex hormones [40]; differences in visceral fat [41], biological variation in haemoglobin glycation; and interindividual differences in red cell 2,3- diphosphoglycerate and in fructosamine 3-kinase [13, 42].

Some studies have been performed looking at the role of ethnicity. In a study of diabetes in the Peruvian population, using HPLC referenced to the NGSP for HbA1c measurement, 40% of people diagnosed with diabetes by HbA1c had normal fasting glucose [43]. There is no reference to haemoglobin variants as an influence on this discrepancy. Variance in Hb1Ac levels has been reported in different groups for the same plasma glucose [38]. The situation is further complicated in the presence of haemoglobinopathies. In 2011, the Korean Diabetes Association included using HbA1c ≥ 6.5% (48 mmol/mol) to diagnose diabetes. In an editorial from Korea, Seo and Lee noted that many studies found that ethnicity influences HbA1c and suggest that the utility of HbA1c may have to be confirmed in different populations [44]. A review of HbA1c as a diabetes screening tool recognises that variant haemoglobins may impact at an individual patient level but could be hidden in a large-scale population study [45]. It is acknowledged that clinically silent Hb variants may be a problem in routine practice [46]. This is particularly relevant when it comes to diagnosing diabetes when analyser precision and other factors affecting A1c have the potential to impact on diagnosis. Accuracy and precision of laboratory analyser performance are particularly important at HbA1c cut-off values of 6.5% (48 mmol/mol) for diabetes diagnosis, at levels in the 5.7–6.4% (46 mmol/mol) range indicating impaired glucose tolerance, and at 5.4% (36 mmol/mol) in gestational diabetes. The IFCC task force on implementation of HbA1c standardisation sets out stringent criteria with a total allowable error set at 0.46% (5 mmol/mol). The best performing method was found to be the Sebi Capillarys 2 Flex Piercing, which is a capillary electrophoresis method which flags up haemoglobinopathies consistently [47].

### Personalised medicine and HbA1c

This is the new paradigm; moving away from race-based medicine and broad categorisations to truly personalised medicine means that we need to consider variants in all patients where discordance is seen between the HbA1c and their glucose levels. While some early studies focus on the association between race and Hb variants, the prevalence of clinically significant variants across the global population is 5.2% [12]. The question of whether patients with diabetes should know their globin chain status arises because of false positive and negative HbA1c results with some laboratory methods. The National Diabetes Information Clearinghouse (NDIC) reminds healthcare providers to suspect a haemoglobinopathy when the HbA1c result is different than expected; the result is greater than 15% (140 mmol/mol); the results of self-monitoring of blood glucose have a low correlation with HbA1c results; and the HbA1c result is different from the previous result following a change in laboratory method. HbA1c should not be used with HbSS, HbCC or HbSC, because of anaemia and increased red cell turnover and transfusion [48]. Individuals with family origins from malaria areas should be considered for haemoglobinopathies.

### Point-of-care testing (POCT) and HbA1c

The issue of haemoglobinopathies in POCT has not been widely considered. A review in clinical chemistry in 2011 made no reference to the issue [49]. Suggested desirable analytical goals for imprecision, bias and total error are 1.3%, 1.9% and 3.9%, respectively [50]. In a sponsored expert review, again no mention is made of the possible impact of haemoglobinopathies [51].

The Diabetes Control and Complications Trial reported that a 10% reduction in HbA1c level resulted in a 43–45% reduction in retinopathy [1]. Assuming a biological variation of 2%, for differentiation of a HbA1c of 7.0% (53 mmol/mol) from 7.6% (60 mmol/mol), an imprecision of 2% CV is required [52]. At present, 3% is a more realistic goal for POCT as the 2% target is not met by laboratory-based immunoassays [53], but ideally analytical goals for POCT and laboratory performance should be identical at ≤ 2% as we have suggested [54]. In a study of eight POCT HbA1c instruments only two—the Afinion (Axis-Shield, Oslo, based on affinity separation) and the DCA Vantage (Siemens Healthcare Diagnostics, Tarrytown, NY, based on latex agglutination)—were able to meet these stringent performance criteria of CV of 3% [55], although the same authors issued an update in 2014 indicating that 4 of 7 instruments met criteria of stringency [56]. An evaluation of In2it from Bio-Rad and DCA Vantage from Siemens showed significant differences dependent on the lot number of the cartridges. At HbA1c levels of 5.1% (32 mmol/mol) and 11.2% (99 mmol/mol), the CVs for In2it were 4.9% and 3.3%, respectively, and 1.7–1.8% and 3.7–5.5% for the DCA Vantage with cartridges of different lot numbers [57]. Rigorous external and internal quality procedures are clearly necessary for patient safety in monitoring or diagnosing diabetes using a POCT device.

### Alternatives to HbA1c in assessing long-term glycaemic control

With many haemoglobinopathies, using HBA1c may be invalid, and alternative monitoring of diabetes control includes fructosamine, also called glycosylated serum protein, glycated serum albumin or self-monitoring of blood glucose. Fructosamine is not standardised, and the relationship with blood glucose levels or complications risk is not established. Notwithstanding these limitations, it is our practice to measure fructosamine in patients with hemoglobinopathies as a next best alternative indicative of medium-term glycaemic control. The use of fructosamine measurement may also provide a guide to the magnitude of the discordance between true glycaemic control and measured HbA1c in haemoglobinopathies. The role of continuous glucose monitoring in patients in whom haemoglobin variants preclude the use of the A1c may need to be expanded.

## Conclusion

We conclude that given the demographic changes in our population, there needs to be greater understanding of factors that impact on HbA1c as it is used for diagnosing and monitoring diabetes. About 1% of patients may have haemoglobin variants that can interfere with the interpretation of the test. Best practice should involve (1) testing all patients to establish their personal haemoglobin subtype before entering life-long monitoring; (2) the use of boronate affinity chromatography methods; (3) the need to establish reference values for diabetes diagnosis and management for individual haemoglobin subtypes [58]; and (4) use of other indicators such as fructosamine or daily multi-glucose levels when HbA1c levels are technically invalid.

The Irish External Quality Assurance Scheme suggests that the following wording be appended to relevant laboratory reports: “Haemoglobin variant detected, interpret HbA1c result with caution. Do not use this result for diagnosis or to assess concordance with glycaemic targets” [59]. The question of haemoglobin variants and point-of-care testing must also be considered. All patients with haemoglobinopathies must be informed that there may be interference with HbA1c measurements, whether testing is laboratory- or point-of-care-based which may increase or decrease HbA1c values. We also propose that healthcare professionals caring for people with diabetes should be alert to the possibility of a haemoglobin variant whenever there appears to be discordance between the HbA1c and other markers of glycaemic control including home blood glucose monitoring and modern technological ways of measuring glucose such as flash or continuous glucose monitoring.
